# 3-(2-Oxo-2,3,4,5-tetra­hydro­furan-3-yl)-1-benzofuran-2-carbonitrile

**DOI:** 10.1107/S1600536812036835

**Published:** 2012-08-31

**Authors:** Kensuke Okuda, Takashi Hirota, Yuta Nishina, Hiroyuki Ishida

**Affiliations:** a Laboratory of Medicinal and Pharmaceutical Chemistry, Gifu Pharmaceutical University, Gifu 501-1196, Japan; b Laboratory of Pharmaceutical Chemistry, Faculty of Pharmaceutical Sciences, Okayama University, Okayama 700-8530, Japan; c Research Core for Interdisciplinary Sciences, Okayama University, Okayama 700-8530, Japan; d Department of Chemistry, Faculty of Science, Okayama University, Okayama 700-8530, Japan

## Abstract

The asymmetric unit of the title compound, C_13_H_9_NO_3_, consists of two crystallographically independent mol­ecules. In each mol­ecule, the tetra­hydro­furan (THF) ring adopts an envelope conformation with one of the methyl­ene C atoms positioned at the flap. The dihedral angles between the mean plane of the THF and the benzofuran ring system are 70.85 (5) and 89.59 (6)°. In the crystal, mol­ecules are stacked in a column along the *a*-axis direction through C—H⋯O hydrogen bonds, with columns further linked by C—H⋯N and C—H⋯O inter­actions.

## Related literature

For a recent report on the development of complex heterocyclic skeletons for potential pharmaceutics in one step using the Truce–Smiles rearrangement, see: Okuda *et al.* (2011 ▶). For the synthesis, see: Okuda *et al.* (2012 ▶).
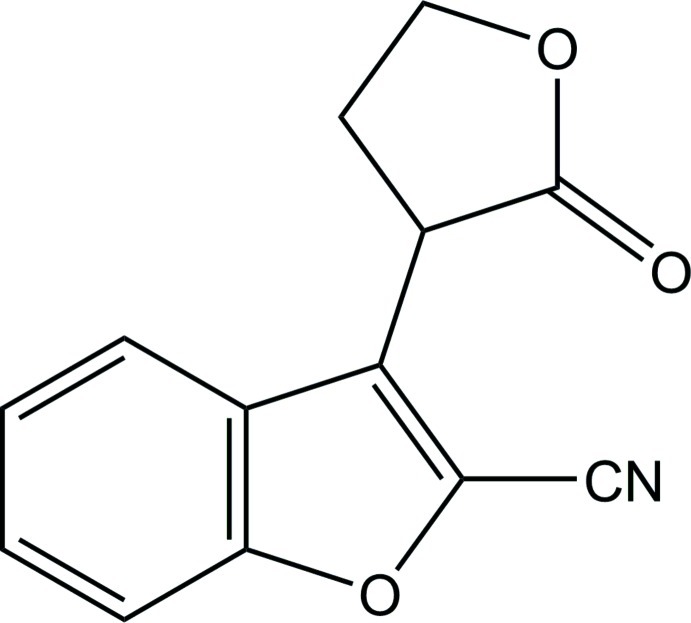


## Experimental

### 

#### Crystal data

C_13_H_9_NO_3_
*M* *_r_* = 227.22
Triclinic, 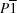

*a* = 5.2724 (7) Å
*b* = 10.7340 (16) Å
*c* = 19.176 (3) Å
α = 82.634 (4)°
β = 82.532 (5)°
γ = 80.371 (4)°
*V* = 1054.6 (3) Å^3^
*Z* = 4
Mo *K*α radiation
μ = 0.10 mm^−1^
*T* = 180 K
0.36 × 0.10 × 0.10 mm

#### Data collection

Rigaku R-AXIS RAPIDII diffractometer
Absorption correction: numerical (*NUMABS*; Higashi, 1999 ▶) *T*_min_ = 0.974, *T*_max_ = 0.990
16333 measured reflections
6117 independent reflections
4415 reflections with *I* > 2σ(*I*)
*R*_int_ = 0.041

#### Refinement

*R*[*F* ^2^ > 2σ(*F* ^2^)] = 0.047
*wR*(*F* ^2^) = 0.133
*S* = 1.11
6117 reflections
307 parameters
H-atom parameters constrained
Δρ_max_ = 0.32 e Å^−3^
Δρ_min_ = −0.27 e Å^−3^

### 

Data collection: *PROCESS-AUTO* (Rigaku/MSC, 2004 ▶); cell refinement: *PROCESS-AUTO*; data reduction: *CrystalStructure* (Rigaku/MSC, 2004 ▶); program(s) used to solve structure: *SHELXS97* (Sheldrick, 2008 ▶); program(s) used to refine structure: *SHELXL97* (Sheldrick, 2008 ▶); molecular graphics: *ORTEP-3* (Farrugia, 1997 ▶); software used to prepare material for publication: *SHELXL97* and *PLATON* (Spek, 2009 ▶).

## Supplementary Material

Crystal structure: contains datablock(s) global, I. DOI: 10.1107/S1600536812036835/gg2098sup1.cif

Structure factors: contains datablock(s) I. DOI: 10.1107/S1600536812036835/gg2098Isup2.hkl

Supplementary material file. DOI: 10.1107/S1600536812036835/gg2098Isup3.cml

Additional supplementary materials:  crystallographic information; 3D view; checkCIF report

## Figures and Tables

**Table 1:** Hydrogen-bond geometry (Å, °)

*D*—H⋯*A*	*D*—H	H⋯*A*	*D*⋯*A*	*D*—H⋯*A*
C4—H4⋯O6^i^	0.95	2.56	3.3422 (17)	140
C10—H10⋯O3^ii^	1.00	2.51	3.3619 (16)	143
C17—H17⋯O6^ii^	0.95	2.46	3.3143 (18)	150
C20—H20⋯N2^iii^	0.95	2.56	3.3936 (19)	146

Symmetry codes: (i) 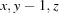; (ii) 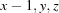; (iii) 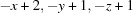.

**Fig. 1.**
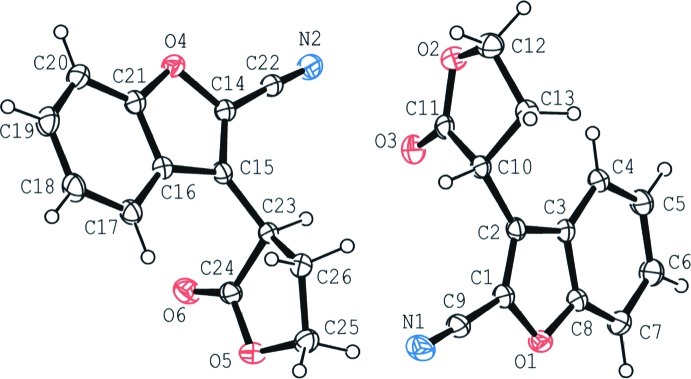
The asymmetric unit of the title compound, with the atom-labeling. Displacement ellipsoids of non-H atoms are drawn at the 50% probability level.

**Fig. 2.**
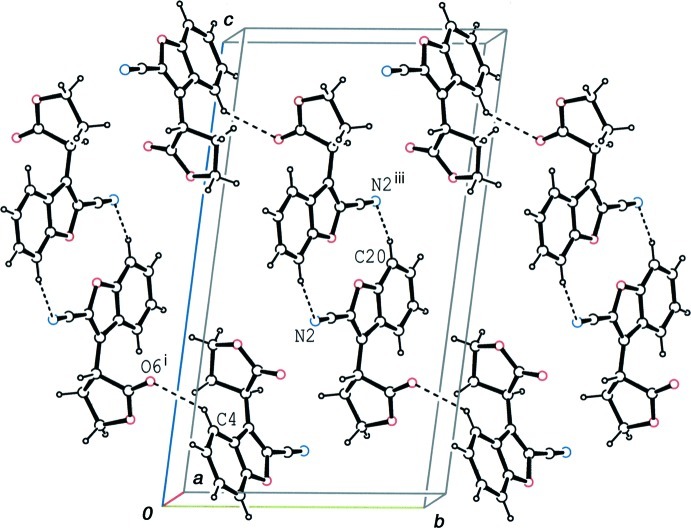
A packing diagram of the title compound, viewed along the a axis. Dashed lines indicate the C—H···O and C—H···N hydrogen bonds. [Symmetry codes: (i) x, y - 1, z; (iii) -x + 2, -y + 1, -z + 1.]

## supplementary crystallographic information

### Comment

As an extension of our work to develop complex heterocyclic skeletons for potential pharmaceutics in one step using the Truce-Smiles rearrangement (Okuda *et al.*, 2011), we have now explored the reaction of 3-(3-ethoxycarbonylpropoxy)[1]benzofuran-2-carbonitrile with bases. In previous work, reaction of 3-(3-cyanopropoxy)benzofuran-2-carbonitrile with potassium *tert*-butoxide in tetrahydrofuran, afforded 5-amino-1,2-dihydro[1]benzofuro[3,2-*d*]furo[2,3-*b*]pyridine (17%, Truce-Smiles rearrangement product) and 5-amino-2,3-dihydro[1]benzofuro[3,2-*b*]oxepin-4-carbonitrile (39%, Thorpe-Ziegler reaction product) (Okuda *et al.*, 2012). In the present study, we have replaced the 3-(3-cyanopropoxy) group with the 3-(3-ethoxycarbonylpropoxy) group. This change of the electrophilic moiety from nitrile to ester produces new interesting rearrangement products. Thus, the reaction of 3-(3-ethoxycarbonylpropoxy)[1]benzofuran-2-carbonitrile with postassium *tert*-butoxide in tetrahydrofuran affords 3-(2-oxo-2,3,4,5-tetrahydrofuran-3-yl)[1]benzofuran-2-carbonitrile (37%, Truce-Smiles rearrangement product) and ethyl 5-amino-2,3-dihydro[1]benzofuro[3,2-*b*]oxepin-4-carboxylate (10%, Thorpe-Ziegler reaction product) (Okuda *et al.*, 2012).

The asymmetric unit of the title compound consists of two crystallographically independent molecules. In the molecules, the tetrahydrofuran C10/C11/O2/C12/C13 and C23/C24/O5/C25/C26 rings adopt envelope conformations with atoms C13 and C26, respectively, at the flaps. The dihedral angle between the mean plane of the tetrahydrofuran C10/C11/O2/C12/C13 ring and the benzofuran C1–C8/O1 ring system is 70.85 (5)°, while the angle between the mean plane of the C23/C24/O5/C25/C26 ring and the C14–C21/O4 ring system is 89.59 (6)°. In the crystal, molecules are stacked in column along the *a* axis through C10—H10···O3^ii^ and C17—H17···O6^ii^ (symmetry code in Table 1) hydrogen bonds. The columns are further linked by C4—H4···O6^i^ and C20—H20···N2^iii^ (symmetry codes in Table 1) hydrogen bonds.

### Experimental

The detailed experimental procedure for the synthesis of 3-(2-oxo-2,3,4,5-tetrahydrofuran-3-yl)-2-carbonitrile (m.p. 378–381 K from cyclohexane) from 3-(3-ethoxycarbonylpropoxy)[1]benzofuran-2-carbonitrile was described in our previous paper (Okuda *et al.*, 2012). Single crystals suitable for X-ray diffraction were obtained from an *n*-hexane/ethyl acetate solution.

### Refinement

H atoms were located in a difference Fourier map and then were positioned geometrically (C—H = 0.95, 0.99 or 1.00 Å) and refined as riding, with *U*_iso_(H) = 1.2*U*_eq_(C).

### Crystal data

**Table:**

C_13_H_9_NO_3_	*Z* = 4
*M**_r_* = 227.22	*F*(000) = 472.00
Triclinic, *P*1	*D*_x_ = 1.431 Mg m^−^^3^
Hall symbol: -P 1	Mo *K*α radiation, λ = 0.71075 Å
*a* = 5.2724 (7) Å	Cell parameters from 12779 reflections
*b* = 10.7340 (16) Å	θ = 3.1–30.1°
*c* = 19.176 (3) Å	µ = 0.10 mm^−^^1^
α = 82.634 (4)°	*T* = 180 K
β = 82.532 (5)°	Block, colourless
γ = 80.371 (4)°	0.36 × 0.10 × 0.10 mm
*V* = 1054.6 (3) Å^3^	

### Data collection

**Table:**

Rigaku R-AXIS RAPIDII diffractometer	4415 reflections with *I* > 2σ(*I*)
Detector resolution: 10.00 pixels mm^-1^	*R*_int_ = 0.041
ω scans	θ_max_ = 30.0°, θ_min_ = 3.1°
Absorption correction: numerical (*NUMABS*; Higashi, 1999)	*h* = −7→7
*T*_min_ = 0.974, *T*_max_ = 0.990	*k* = −15→15
16333 measured reflections	*l* = −26→25
6117 independent reflections	

### Refinement

**Table:**

Refinement on *F*^2^	Primary atom site location: structure-invariant direct methods
Least-squares matrix: full	Secondary atom site location: difference Fourier map
*R*[*F*^2^ > 2σ(*F*^2^)] = 0.047	Hydrogen site location: inferred from neighbouring sites
*wR*(*F*^2^) = 0.133	H-atom parameters constrained
*S* = 1.11	*w* = 1/[σ^2^(*F*_o_^2^) + (0.0663*P*)^2^ + 0.0901*P*] where *P* = (*F*_o_^2^ + 2*F*_c_^2^)/3
6117 reflections	(Δ/σ)_max_ < 0.001
307 parameters	Δρ_max_ = 0.32 e Å^−^^3^
0 restraints	Δρ_min_ = −0.27 e Å^−^^3^

### Special details

**Table:**

Geometry. All e.s.d.'s (except the e.s.d. in the dihedral angle between two l.s. planes) are estimated using the full covariance matrix. The cell e.s.d.'s are taken into account individually in the estimation of e.s.d.'s in distances, angles and torsion angles; correlations between e.s.d.'s in cell parameters are only used when they are defined by crystal symmetry. An approximate (isotropic) treatment of cell e.s.d.'s is used for estimating e.s.d.'s involving l.s. planes.
Refinement. Refinement of *F*^2^ against ALL reflections. The weighted *R*-factor *wR* and goodness of fit *S* are based on *F*^2^, conventional *R*-factors *R* are based on *F*, with *F* set to zero for negative *F*^2^. The threshold expression of *F*^2^ > σ(*F*^2^) is used only for calculating *R*-factors(gt) *etc*. and is not relevant to the choice of reflections for refinement. *R*-factors based on *F*^2^ are statistically about twice as large as those based on *F*, and *R*- factors based on ALL data will be even larger.

### Fractional atomic coordinates and isotropic or equivalent isotropic displacement parameters (Å^2^)

**Table:**

	*x*	*y*	*z*	*U*_iso_*/*U*_eq_	
O1	0.58378 (18)	0.34215 (9)	0.03832 (5)	0.0323 (2)	
O2	0.69558 (19)	0.15370 (9)	0.32831 (5)	0.0361 (2)	
O3	0.81903 (19)	0.32278 (10)	0.26050 (5)	0.0379 (2)	
O4	0.73089 (18)	0.58516 (9)	0.45824 (5)	0.0324 (2)	
O5	0.60788 (19)	0.78752 (9)	0.16810 (5)	0.0356 (2)	
O6	0.8676 (2)	0.82575 (9)	0.24258 (5)	0.0380 (2)	
N1	0.0379 (3)	0.50838 (13)	0.11992 (7)	0.0445 (3)	
N2	1.2131 (2)	0.39902 (12)	0.36460 (6)	0.0394 (3)	
C1	0.4424 (2)	0.34060 (12)	0.10448 (7)	0.0295 (3)	
C2	0.5470 (2)	0.24973 (11)	0.15304 (7)	0.0266 (2)	
C3	0.7757 (2)	0.18596 (11)	0.11503 (6)	0.0265 (3)	
C4	0.9699 (3)	0.08551 (12)	0.13326 (7)	0.0294 (3)	
H4	0.9624	0.0405	0.1793	0.035*	
C5	1.1724 (3)	0.05397 (13)	0.08222 (7)	0.0353 (3)	
H5	1.3072	−0.0133	0.0936	0.042*	
C6	1.1836 (3)	0.11914 (14)	0.01379 (8)	0.0378 (3)	
H6	1.3263	0.0951	−0.0199	0.045*	
C7	0.9925 (3)	0.21716 (13)	−0.00570 (7)	0.0347 (3)	
H7	0.9987	0.2614	−0.0519	0.042*	
C8	0.7911 (3)	0.24714 (12)	0.04626 (7)	0.0290 (3)	
C9	0.2170 (3)	0.43326 (13)	0.11173 (7)	0.0333 (3)	
C10	0.4607 (2)	0.22821 (11)	0.23048 (6)	0.0263 (2)	
H10	0.3051	0.2922	0.2424	0.032*	
C11	0.6764 (2)	0.24452 (12)	0.27295 (7)	0.0286 (3)	
C12	0.4900 (3)	0.07752 (15)	0.33328 (8)	0.0404 (3)	
H12A	0.3455	0.1071	0.3686	0.048*	
H12B	0.5549	−0.0130	0.3473	0.048*	
C13	0.4013 (3)	0.09468 (12)	0.26003 (7)	0.0315 (3)	
H13A	0.2141	0.0906	0.2626	0.038*	
H13B	0.4994	0.0293	0.2307	0.038*	
C14	0.8037 (2)	0.56118 (12)	0.38884 (7)	0.0283 (3)	
C15	0.6447 (2)	0.63010 (11)	0.34307 (7)	0.0261 (2)	
C16	0.4503 (2)	0.70580 (11)	0.38674 (7)	0.0271 (3)	
C17	0.2297 (3)	0.79571 (12)	0.37446 (7)	0.0327 (3)	
H17	0.1802	0.8192	0.3281	0.039*	
C18	0.0872 (3)	0.84879 (14)	0.43205 (8)	0.0386 (3)	
H18	−0.0630	0.9094	0.4249	0.046*	
C19	0.1582 (3)	0.81571 (15)	0.50069 (8)	0.0405 (3)	
H19	0.0557	0.8549	0.5388	0.049*	
C20	0.3731 (3)	0.72762 (14)	0.51429 (7)	0.0366 (3)	
H20	0.4220	0.7043	0.5607	0.044*	
C21	0.5136 (3)	0.67511 (12)	0.45587 (7)	0.0301 (3)	
C22	1.0308 (3)	0.47067 (13)	0.37642 (7)	0.0306 (3)	
C23	0.6739 (2)	0.62988 (11)	0.26448 (6)	0.0264 (3)	
H23	0.8214	0.5626	0.2511	0.032*	
C24	0.7321 (2)	0.75716 (12)	0.22642 (7)	0.0284 (3)	
C25	0.4614 (3)	0.68768 (14)	0.15984 (7)	0.0342 (3)	
H25A	0.5536	0.6343	0.1232	0.041*	
H25B	0.2881	0.7249	0.1459	0.041*	
C26	0.4373 (3)	0.60905 (13)	0.23163 (7)	0.0315 (3)	
H26A	0.4425	0.5181	0.2264	0.038*	
H26B	0.2748	0.6398	0.2606	0.038*	

### Atomic displacement parameters (Å^2^)

**Table:**

	*U*^11^	*U*^22^	*U*^33^	*U*^12^	*U*^13^	*U*^23^
O1	0.0354 (5)	0.0305 (5)	0.0270 (5)	0.0052 (4)	−0.0027 (4)	−0.0016 (4)
O2	0.0398 (5)	0.0368 (5)	0.0322 (5)	−0.0040 (4)	−0.0094 (4)	−0.0020 (4)
O3	0.0365 (5)	0.0360 (5)	0.0432 (6)	−0.0076 (4)	−0.0034 (4)	−0.0105 (4)
O4	0.0361 (5)	0.0327 (5)	0.0255 (5)	0.0058 (4)	−0.0050 (4)	−0.0055 (4)
O5	0.0406 (5)	0.0344 (5)	0.0303 (5)	−0.0024 (4)	−0.0094 (4)	0.0032 (4)
O6	0.0402 (6)	0.0323 (5)	0.0423 (6)	−0.0081 (4)	−0.0082 (4)	0.0003 (4)
N1	0.0423 (7)	0.0399 (7)	0.0425 (7)	0.0112 (6)	0.0003 (5)	0.0002 (5)
N2	0.0389 (6)	0.0426 (7)	0.0328 (6)	0.0092 (5)	−0.0057 (5)	−0.0081 (5)
C1	0.0299 (6)	0.0269 (6)	0.0299 (6)	0.0020 (5)	−0.0016 (5)	−0.0057 (5)
C2	0.0269 (6)	0.0231 (5)	0.0292 (6)	0.0005 (4)	−0.0022 (5)	−0.0073 (5)
C3	0.0281 (6)	0.0239 (6)	0.0270 (6)	−0.0001 (5)	−0.0010 (5)	−0.0075 (5)
C4	0.0320 (6)	0.0252 (6)	0.0293 (6)	0.0015 (5)	−0.0028 (5)	−0.0047 (5)
C5	0.0337 (7)	0.0308 (7)	0.0371 (7)	0.0066 (5)	−0.0001 (5)	−0.0069 (5)
C6	0.0365 (7)	0.0383 (7)	0.0343 (7)	0.0034 (6)	0.0047 (6)	−0.0091 (6)
C7	0.0387 (7)	0.0351 (7)	0.0271 (6)	0.0003 (6)	0.0017 (5)	−0.0050 (5)
C8	0.0309 (6)	0.0257 (6)	0.0290 (6)	0.0018 (5)	−0.0035 (5)	−0.0053 (5)
C9	0.0350 (7)	0.0307 (6)	0.0314 (7)	0.0020 (5)	−0.0031 (5)	−0.0028 (5)
C10	0.0249 (6)	0.0252 (6)	0.0270 (6)	0.0022 (4)	−0.0008 (4)	−0.0062 (5)
C11	0.0299 (6)	0.0264 (6)	0.0283 (6)	0.0027 (5)	−0.0007 (5)	−0.0098 (5)
C12	0.0474 (9)	0.0386 (8)	0.0351 (8)	−0.0102 (6)	−0.0044 (6)	0.0007 (6)
C13	0.0311 (7)	0.0292 (6)	0.0336 (7)	−0.0038 (5)	−0.0019 (5)	−0.0044 (5)
C14	0.0312 (6)	0.0261 (6)	0.0266 (6)	0.0006 (5)	−0.0035 (5)	−0.0051 (5)
C15	0.0275 (6)	0.0216 (5)	0.0282 (6)	−0.0008 (4)	−0.0021 (5)	−0.0041 (4)
C16	0.0299 (6)	0.0237 (6)	0.0274 (6)	−0.0018 (5)	−0.0029 (5)	−0.0051 (5)
C17	0.0328 (7)	0.0280 (6)	0.0367 (7)	0.0015 (5)	−0.0064 (5)	−0.0062 (5)
C18	0.0334 (7)	0.0335 (7)	0.0468 (8)	0.0076 (6)	−0.0049 (6)	−0.0121 (6)
C19	0.0416 (8)	0.0401 (8)	0.0377 (8)	0.0027 (6)	0.0027 (6)	−0.0153 (6)
C20	0.0417 (8)	0.0384 (7)	0.0279 (6)	0.0009 (6)	−0.0017 (5)	−0.0084 (5)
C21	0.0318 (7)	0.0274 (6)	0.0299 (6)	0.0006 (5)	−0.0029 (5)	−0.0055 (5)
C22	0.0334 (7)	0.0307 (6)	0.0261 (6)	0.0006 (5)	−0.0044 (5)	−0.0037 (5)
C23	0.0296 (6)	0.0234 (6)	0.0249 (6)	0.0021 (5)	−0.0039 (5)	−0.0045 (4)
C24	0.0275 (6)	0.0278 (6)	0.0272 (6)	0.0021 (5)	−0.0019 (5)	−0.0024 (5)
C25	0.0338 (7)	0.0381 (7)	0.0311 (7)	−0.0014 (6)	−0.0080 (5)	−0.0063 (6)
C26	0.0354 (7)	0.0293 (6)	0.0307 (7)	−0.0038 (5)	−0.0056 (5)	−0.0060 (5)

### Geometric parameters (Å, º)

**Table:**

O1—C8	1.3741 (15)	C10—H10	1.0000
O1—C1	1.3850 (15)	C12—C13	1.5169 (19)
O2—C11	1.3480 (16)	C12—H12A	0.9900
O2—C12	1.4500 (18)	C12—H12B	0.9900
O3—C11	1.2005 (16)	C13—H13A	0.9900
O4—C21	1.3706 (15)	C13—H13B	0.9900
O4—C14	1.3809 (15)	C14—C15	1.3555 (17)
O5—C24	1.3458 (16)	C14—C22	1.4251 (17)
O5—C25	1.4568 (17)	C15—C16	1.4440 (17)
O6—C24	1.2003 (16)	C15—C23	1.4952 (17)
N1—C9	1.1427 (18)	C16—C21	1.3943 (18)
N2—C22	1.1434 (17)	C16—C17	1.4059 (17)
C1—C2	1.3576 (18)	C17—C18	1.3825 (19)
C1—C9	1.4207 (17)	C17—H17	0.9500
C2—C3	1.4411 (16)	C18—C19	1.401 (2)
C2—C10	1.4958 (17)	C18—H18	0.9500
C3—C8	1.3945 (18)	C19—C20	1.379 (2)
C3—C4	1.3998 (17)	C19—H19	0.9500
C4—C5	1.3804 (18)	C20—C21	1.3886 (18)
C4—H4	0.9500	C20—H20	0.9500
C5—C6	1.405 (2)	C23—C24	1.5232 (18)
C5—H5	0.9500	C23—C26	1.5282 (17)
C6—C7	1.3799 (19)	C23—H23	1.0000
C6—H6	0.9500	C25—C26	1.5220 (18)
C7—C8	1.3845 (18)	C25—H25A	0.9900
C7—H7	0.9500	C25—H25B	0.9900
C10—C11	1.5272 (17)	C26—H26A	0.9900
C10—C13	1.5392 (18)	C26—H26B	0.9900
			
C8—O1—C1	104.64 (9)	C10—C13—H13B	111.2
C11—O2—C12	110.37 (10)	H13A—C13—H13B	109.1
C21—O4—C14	104.81 (9)	C15—C14—O4	113.40 (11)
C24—O5—C25	110.35 (10)	C15—C14—C22	130.44 (12)
C2—C1—O1	113.40 (11)	O4—C14—C22	116.15 (11)
C2—C1—C9	130.13 (12)	C14—C15—C16	104.75 (11)
O1—C1—C9	116.46 (11)	C14—C15—C23	126.85 (11)
C1—C2—C3	104.72 (11)	C16—C15—C23	128.36 (11)
C1—C2—C10	127.92 (11)	C21—C16—C17	118.56 (12)
C3—C2—C10	127.11 (11)	C21—C16—C15	106.28 (11)
C8—C3—C4	119.08 (11)	C17—C16—C15	135.16 (12)
C8—C3—C2	106.54 (11)	C18—C17—C16	117.81 (13)
C4—C3—C2	134.36 (12)	C18—C17—H17	121.1
C5—C4—C3	117.89 (12)	C16—C17—H17	121.1
C5—C4—H4	121.1	C17—C18—C19	121.84 (13)
C3—C4—H4	121.1	C17—C18—H18	119.1
C4—C5—C6	121.44 (12)	C19—C18—H18	119.1
C4—C5—H5	119.3	C20—C19—C18	121.58 (13)
C6—C5—H5	119.3	C20—C19—H19	119.2
C7—C6—C5	121.68 (12)	C18—C19—H19	119.2
C7—C6—H6	119.2	C19—C20—C21	115.81 (13)
C5—C6—H6	119.2	C19—C20—H20	122.1
C6—C7—C8	115.93 (12)	C21—C20—H20	122.1
C6—C7—H7	122.0	O4—C21—C20	124.84 (12)
C8—C7—H7	122.0	O4—C21—C16	110.75 (11)
O1—C8—C7	125.34 (12)	C20—C21—C16	124.40 (12)
O1—C8—C3	110.67 (11)	N2—C22—C14	178.21 (14)
C7—C8—C3	123.96 (12)	C15—C23—C24	111.65 (10)
N1—C9—C1	177.51 (15)	C15—C23—C26	116.74 (10)
C2—C10—C11	109.82 (10)	C24—C23—C26	102.94 (10)
C2—C10—C13	117.21 (10)	C15—C23—H23	108.4
C11—C10—C13	102.61 (10)	C24—C23—H23	108.4
C2—C10—H10	108.9	C26—C23—H23	108.4
C11—C10—H10	108.9	O6—C24—O5	121.30 (12)
C13—C10—H10	108.9	O6—C24—C23	128.18 (12)
O3—C11—O2	122.11 (12)	O5—C24—C23	110.52 (11)
O3—C11—C10	127.51 (12)	O5—C25—C26	105.93 (10)
O2—C11—C10	110.35 (11)	O5—C25—H25A	110.5
O2—C12—C13	105.67 (11)	C26—C25—H25A	110.5
O2—C12—H12A	110.6	O5—C25—H25B	110.5
C13—C12—H12A	110.6	C26—C25—H25B	110.5
O2—C12—H12B	110.6	H25A—C25—H25B	108.7
C13—C12—H12B	110.6	C25—C26—C23	103.01 (11)
H12A—C12—H12B	108.7	C25—C26—H26A	111.2
C12—C13—C10	102.80 (11)	C23—C26—H26A	111.2
C12—C13—H13A	111.2	C25—C26—H26B	111.2
C10—C13—H13A	111.2	C23—C26—H26B	111.2
C12—C13—H13B	111.2	H26A—C26—H26B	109.1
			
C8—O1—C1—C2	−0.64 (15)	C21—O4—C14—C15	−0.31 (15)
C8—O1—C1—C9	178.45 (12)	C21—O4—C14—C22	179.12 (12)
O1—C1—C2—C3	−0.40 (15)	O4—C14—C15—C16	−0.08 (15)
C9—C1—C2—C3	−179.32 (14)	C22—C14—C15—C16	−179.42 (14)
O1—C1—C2—C10	174.12 (12)	O4—C14—C15—C23	177.84 (11)
C9—C1—C2—C10	−4.8 (2)	C22—C14—C15—C23	−1.5 (2)
C1—C2—C3—C8	1.26 (14)	C14—C15—C16—C21	0.44 (14)
C10—C2—C3—C8	−173.32 (12)	C23—C15—C16—C21	−177.44 (12)
C1—C2—C3—C4	179.54 (14)	C14—C15—C16—C17	−179.35 (15)
C10—C2—C3—C4	5.0 (2)	C23—C15—C16—C17	2.8 (2)
C8—C3—C4—C5	1.69 (19)	C21—C16—C17—C18	−0.1 (2)
C2—C3—C4—C5	−176.42 (14)	C15—C16—C17—C18	179.70 (14)
C3—C4—C5—C6	−0.7 (2)	C16—C17—C18—C19	0.3 (2)
C4—C5—C6—C7	−0.3 (2)	C17—C18—C19—C20	−0.5 (2)
C5—C6—C7—C8	0.2 (2)	C18—C19—C20—C21	0.3 (2)
C1—O1—C8—C7	−176.97 (13)	C14—O4—C21—C20	179.82 (13)
C1—O1—C8—C3	1.47 (14)	C14—O4—C21—C16	0.60 (14)
C6—C7—C8—O1	179.11 (13)	C19—C20—C21—O4	−179.19 (13)
C6—C7—C8—C3	0.9 (2)	C19—C20—C21—C16	−0.1 (2)
C4—C3—C8—O1	179.67 (11)	C17—C16—C21—O4	179.17 (12)
C2—C3—C8—O1	−1.74 (14)	C15—C16—C21—O4	−0.67 (15)
C4—C3—C8—C7	−1.9 (2)	C17—C16—C21—C20	0.0 (2)
C2—C3—C8—C7	176.72 (13)	C15—C16—C21—C20	−179.88 (13)
C1—C2—C10—C11	−118.27 (14)	C14—C15—C23—C24	−113.20 (14)
C3—C2—C10—C11	55.08 (16)	C16—C15—C23—C24	64.24 (16)
C1—C2—C10—C13	125.22 (14)	C14—C15—C23—C26	128.77 (14)
C3—C2—C10—C13	−61.43 (17)	C16—C15—C23—C26	−53.79 (17)
C12—O2—C11—O3	176.41 (12)	C25—O5—C24—O6	178.87 (12)
C12—O2—C11—C10	−5.30 (14)	C25—O5—C24—C23	−0.89 (14)
C2—C10—C11—O3	39.87 (17)	C15—C23—C24—O6	38.50 (18)
C13—C10—C11—O3	165.23 (13)	C26—C23—C24—O6	164.52 (14)
C2—C10—C11—O2	−138.30 (10)	C15—C23—C24—O5	−141.76 (11)
C13—C10—C11—O2	−12.94 (13)	C26—C23—C24—O5	−15.74 (13)
C11—O2—C12—C13	21.84 (15)	C24—O5—C25—C26	17.46 (14)
O2—C12—C13—C10	−28.54 (14)	O5—C25—C26—C23	−26.15 (13)
C2—C10—C13—C12	145.02 (11)	C15—C23—C26—C25	147.42 (11)
C11—C10—C13—C12	24.64 (12)	C24—C23—C26—C25	24.76 (12)

### Hydrogen-bond geometry (Å, º)

**Table:**

*D*—H···*A*	*D*—H	H···*A*	*D*···*A*	*D*—H···*A*
C4—H4···O6^i^	0.95	2.56	3.3422 (17)	140
C10—H10···O3^ii^	1.00	2.51	3.3619 (16)	143
C17—H17···O6^ii^	0.95	2.46	3.3143 (18)	150
C20—H20···N2^iii^	0.95	2.56	3.3936 (19)	146

Symmetry codes: (i) *x*, *y*−1, *z*; (ii) *x*−1, *y*, *z*; (iii) −*x*+2, −*y*+1, −*z*+1.
